# Improving Facial Emotion Recognition in Schizophrenia: a Controlled Study Comparing Specific and Attentional Focused Cognitive Remediation

**DOI:** 10.3389/fpsyt.2016.00105

**Published:** 2016-06-21

**Authors:** Baptiste Gaudelus, Jefferson Virgile, Sabrina Geliot, The GAÏA/RECOS Study Team, M. Dupuis, Nicolas Franck

**Affiliations:** ^1^Service Universitaire de Réhabilitation, CL3R, Le Vinatier Hospital, Bron, France; ^2^Département de Réhabilitation Psycho-sociale, St Cyr au Mont d’Or Hospital, St Cyr au Mont d’Or, France; ^3^Centre régional de dépistage et de prise en charge des troubles psychiatriques d’origine génétique, Le Vinatier Hospital, Bron, France; ^4^CRISALID, Clermont de l’Oise Hospital (CHI), Clermont de l’Oise, France; ^5^Unité de Soins Psycho-Sociaux, HDJ S17-S18, C3RP, Sainte-Anne Hospital, Paris, France; ^6^C3R Grenoble, Centre référent de réhabilitation psychosociale, Alpes-Isère Hospital (CHAI), St Egrève, France; ^7^Lyon 1 Claude Bernard University, Lyon, France; ^8^UMR 5229, Centre National de la Recherche Scientifique (CNRS), Lyon, France

**Keywords:** Schizophrenia, social cognition, facial emotions, cognitive remediation, nursing practice

## Abstract

Cognitive impairments associated with schizophrenia are very frequent. They concern both neurocognition and social cognition, including facial emotion recognition. These impairments have a negative impact on the daily functioning, in particular the social and vocational rehabilitation of people with schizophrenia. Previous studies in this area clearly demonstrated the interest of cognitive remediation to improve neurocognitive and social cognitive functioning in schizophrenia. They also established clear links between facial emotion recognition skills and attentional processes. The present study compares the GAÏA s-face program (GAÏA arm), which focuses on facial emotion recognition processes, with the RECOS program (RECOS arm), a neurocognitive remediation therapy focusing on selective attention. Forty people with schizophrenia were randomly distributed between each study arm and assessed pre- (T1) and post- (T2) therapy. The single-blind assessment focused on facial emotion recognition (the main criteria), symptoms, social and subjective functioning, and neurocognitive and social cognitive performance. Both programs were conducted by nurses after a 3-day training session. The study showed a significant improvement in facial emotion recognition performance in both groups, with a significantly larger effect in the GAÏA arm. Symptoms and social functioning also improved in the GAÏA arm, and certain neurocognitive and social cognitive processes improved in both study arms. Further studies are recommended, with larger population samples and a follow-up assessing the long-term preservation of these improvements.

## Introduction

Cognitive impairments associated with schizophrenia affect about four out of five patients. They significantly impact functional recovery, and the social and vocational rehabilitation of people with schizophrenia (1–3). They may also impact all cognitive processes, i.e., neurocognition (memory, attention, executive functions, and processing speed) and social cognition (4–6).

Social cognition can be defined as the ability to construct mental representations about others, oneself and one’s relationships to others (5), and covers several processes. Five components of social cognition are mainly impaired in schizophrenia (6–9): theory of mind, attributional style, social perception, social knowledge, and the perception of emotions on faces and in prosody (i.e., the emotional information provided by variations in pitch, loudness, and speech flow).

The ability to understand the emotional state of others is, therefore, a key aspect of social cognition. Its function is to help adapt one’s behavior according to the “signs” other people give off. The misinterpretation of other people’s emotions or poor communication with one’s own emotions negatively impacts relationships, and leads to difficulty in joining social groups. Impaired emotion perception and expression in schizophrenia have been described since Bleuler (1911) (10), and facial emotion recognition deficit is now clearly identified (11). Various studies showed that this deficit was a trait factor (12) and was observed with less intensity in family members not affected by the disease (13). Although facial affect recognition deficit is not specific to schizophrenia, its frequency and intensity are higher in schizophrenia than in other psychiatric disorders. Some authors suggested that facial affect recognition deficits might be a marker of vulnerability to the disease (14).

The origin of facial emotion processing deficits associated with schizophrenia is still not completely understood but is thought to result from the alteration of various processes.

Several authors (15–17) reported correlations between attention deficit and facial emotion recognition deficit. These correlations are supported by many neuroimaging studies that showing hypoactivity in the frontal cortex in patients with schizophrenia (18); the frontal cortex controls both executive and attentional skills, and is also involved in facial emotion recognition (19).

Other studies focused on patterns of visual attention to faces (20, 21): compared to healthy controls, people with schizophrenia showed reduced number and range of visual saccades, increased duration of fixation, and reduced attention to relevant features during emotion recognition tests.

Facial emotion recognition deficit is also frequently correlated with other difficulties in emotional information processing, especially the production of facial emotions (22, 23), and the processing of affective prosody (24). This may be caused by a deficit in a specific cognitive emotional processing module. This hypothesis is also supported by neuroimaging studies showing that the same brain regions are partly involved in the processing of facial and prosodic emotional information, most notably the upper right temporal cortex, the limbic system, and the prefrontal cortex (25, 26).

Recent research has explored the hypothesis of a gap in the processing of all facial information, including non-emotional information. In people with schizophrenia, such deficit may impair the ability to process configural information (i.e., the physical characteristics making up a face and the relations between its components), which can be common to all faces (i.e., first-order configural information) or specific to one face (i.e., second-order configural information). Maurer et al. (27) proposed a distinction between two processing methods operating at the same time: the holistic processing, in which facial information is processed as a whole, and the componential processing that involves a feature-based analysis relying on second-order configural information processing (28). The impact of impaired configural information processing on facial emotion recognition deficit in people with schizophrenia is still poorly understood. However, the differences reported by various studies between patients with schizophrenia and healthy controls (14, 29) call for further investigation in this particular area.

Although the processes underlying social cognition disorders are not completely understood, cognitive remediation programs targeting social cognition or some of its components (especially theory of mind and the perception of facial emotions) have been recently developed (30–32). Cognitive remediation seems to be the most promising intervention to improve social cognition abilities and especially facial emotion recognition (32), whereas antipsychotic treatments have little effect on such processes (33). Most social cognitive remediation programs are group oriented and based on “standardized” exercises, which may be detrimental to patients. It also makes it more difficult to adapt strategies to each participant’s cognitive and clinical profile. Furthermore, groups do not facilitate individualized homework tasks, which are designed to help transfer therapeutic strategies into daily life.

In the field of neurocognition, recently, several computer-assisted cognitive remediation programs have been developed (34–37). Computer-assisted programs give immediate feedback on the participant’s performance and adjust both the difficulty of the exercises and reinforcement methods (38). Furthermore, it seems that prolonged computer stimulation encourages neural plasticity and the learning of new coping strategies, which are central to cognitive remediation therapy (39). Finally, individual cognitive remediation provides exercises adapted to the participant’s cognitive profile and functional goals (40), and takes into account the metacognitive difficulties associated with schizophrenia (41–43).

In the field of social cognition, the use of computer-assisted methods seems just as relevant, especially to improve emotion recognition. The advantage of this technology lies in the control of all the processes at play in social interactions, and the development of progressive training. Furthermore, it provides a secure environment where the person can practice without risking negative repercussions in real life or generating much anxiety. Using a virtual environment also limits the bias of attributing emotions to others according to the participant’s own emotions. However, the presence of a collaborative therapist remains essential, especially to encourage the transfer and adaptation of strategies into real-life situations (40). Furthermore, the role of the therapist also includes encouraging the participant formulate concrete objectives (increasing, therefore, motivation), and developing individualized exercises to achieve these goals throughout therapy; these exercises will then be put into practice during and outside the sessions.

According to these data, both neurocognitive remediation, focusing on attentional processes, and social cognition remediation, focusing on emotional perception, should increase facial affect recognition in people with schizophrenia. From a clinical perspective, it would be interesting to compare the effects of these strategies on symptoms, and cognitive and social functioning.

The GAÏA s-face (Schizophrenia- Facial Affects recognition Cognitive Enhancement) program (44, 45) is an individual, computer-assisted cognitive remediation therapy focusing on facial emotion recognition. The present controlled efficacy study compares the GAÏA s-face program with the RECOS program, an individual, computer-assisted neurocognitive remediation therapy focusing on attentional processes (46, 47).

## Methods

### Study Design

Clinically stable people with schizophrenia, according to the DSM-IV-TR criteria (48), were recruited in four psychiatric centers in France. They were randomly distributed between two active treatment arms, namely GAÏA and RECOS. The assessments were blind to group allocation and carried out at baseline (T1 = week 0) and post-treatment (T2 = week 11).

The main treatment outcome measure was the TREF (facial emotion recognition test) total score (49).

Secondary measures included clinical ratings, neurocognitive and social cognitive measures, and psychosocial evaluation.

The study was approved by local ethics authority (CPP Lyon Sud–Est VI, project no. AU 940), and declared to the national authority (ANSM: project no. 2011-A00793-38) and on clinicaltrials.gov (project ID: NCT01607424).

After a complete description of the study objectives and procedures, each participant signed a written informed consent.

The two arms of this parallel-group randomized clinical trial consisted of active and comparable interventions in terms of number of sessions, but also in terms of therapist roles and material used. This constitutes an ethical choice, since each participant was provided with an intervention designed to improve his/her cognitive performance and social and professional integration (3, 7, 50). The study design also makes it possible to observe and compare the specific effects of each program on the various cognitive and functional outcomes.

Both interventions were carried out by nurses (except one occupational therapist in the RECOS arm) who all attended a specific 3-day training session. Each nurse was involved in a single arm of the study to ensure equal motivation and investment among therapists for both programs. The possibility to assess the nurses’ skills in providing effective individual cognitive remediation interventions might represent a cost-efficient solution to implement cognitive remediation in the routine care of schizophrenia, for the benefit of a larger number of people.

### Participants

**Figure 1:**
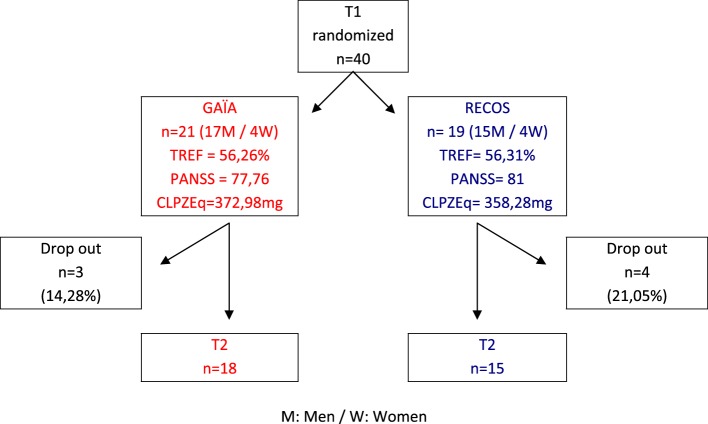
**Group allocation**.

Forty in- and out-patients with stable schizophrenia were included in the trial (see Figure 1). Inclusion criteria were as follows: DSM-IV-TR criteria for schizophrenia (48), with clinical stability confirmed after examination by the psychiatrist-investigator from the recruiting department; age 18–45; impairment of facial affect recognition confirmed by a TREF test score below 69% (SD = −1) (49); native French speakers.

Exclusion criteria included history of neurological illness or trauma; use of somatic medication with cerebral or psychological impact; alcohol or drug addiction (except tobacco); and other cognitive remediation therapy during the period of inclusion in the study.

At T1, there was no significant difference between the GAÏA and RECOS groups in age, gender, symptom intensity measured with the Positive And Negative Symptoms Scale [PANSS (51)], medication doses converted into chlorpromazine equivalent (52), or facial emotion recognition skills measured with the TREF (see Table 1).

**Table 1:** **Group comparison after randomization**.

Test/measure	GAÏA arm	RECOS arm	*p*-value
*n* allocation	21	19	
Gender ratio (w/m)	0.19	0.21	0.89 (ns)
Age	31.71	33.74	0.37 (ns)
TREF total score	55.67%	54.17%	0.63 (ns)
PANSS total score	76.62	81	0.45 (ns)
Positive subscale	16.52	15.26	0.31 (ns)
Negative subscale	20.61	23.37	0.19 (ns)
Medication (chlorpromazine equivalent)	377.83 mg	335.52 mg	0.56 (ns)

### Interventions

**Figure 2:**
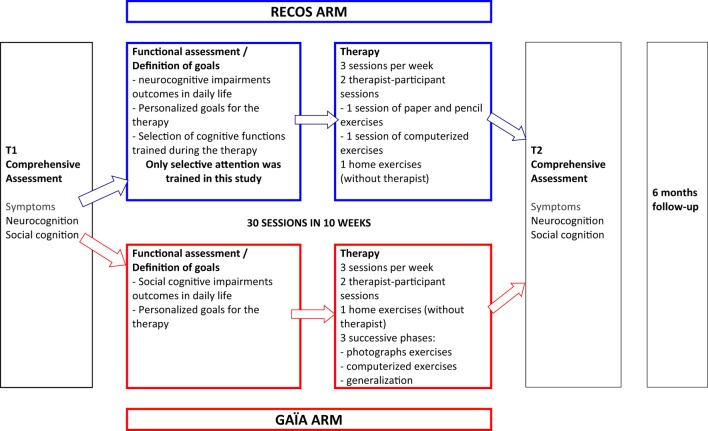
**Steps of GAÏA and RECOS interventions**.

The participants were assessed before (T1) and after (T2) intervention. However, both RECOS and GAÏA s-face programs recommend a 6-month follow-up phase, with a 30-min meeting every 2 weeks between the participant and the therapist (see Figure 2). This meeting, during which no cognitive remediation exercises are performed, is aimed at reinforcing the functional outcomes of cognitive remediation (45, 53).

#### RECOS (Control Arm)

RECOS is an individual neurocognitive remediation therapy with proven effectiveness (35). In its usual form (53), it targets one to three out of six neurocognitive functions (verbal memory, working memory, executive functions, memory and visuo–spatial attention, selective attention, and processing speed), according to the functional implications of their impairments and the participant’s cognitive and clinical profile. The RECOS program comes after a comprehensive assessment of cognitive functions, clinical symptoms, and social functioning. The therapy consists of three 1-h sessions per week, always alternating paper-and-pencil sessions, computer sessions, and home (without therapist) exercises designed to encourage functional benefits.

In the present study, all the patients working with RECOS were trained on selective attention, since it was demonstrated that attentional deficits were linked to facial emotion processing deficits (15–17). All the participants randomly assigned to the RECOS group attended 30 sessions during a 10-week-long treatment.

#### GAÏA s–Face (Experimental Arm)

GAÏA s-face (44, 45) is an individual, social cognitive remediation program focusing on facial emotion processing. The GAÏA program comes after a comprehensive assessment of neurocognition, social cognition, clinical symptoms, and social functioning. The therapy is spread over 30 sessions: three 1-h sessions per week for 10 weeks. One of these sessions (called the transfer session) is performed without the therapist in the participant’s everyday environment. The GAÏA program is divided into three phases: (1) an exercise phase with photographs, aiming to develop strategies to recognize and discriminate joy, anger, and sadness. (2) A computerized exercise phase using videos, to adapt the strategies developed on photographs to dynamic situations with five levels of increasing difficulty. (3) A generalization phase, consisting of at least five therapist–participant sessions and further computerized exercises determined according to the participant’s profile and requests, to recognize and discriminate other basic emotions (fear, disgust, and contempt) and to work on complex emotions.

### Assessment

#### Main Outcome Measure

The main outcome was measured with the TREF (Facial Emotions Recognition Task) total score (49).

The TREF consists of 54 photographs of six male and female faces expressing disgust, contempt, fear, anger, sadness, and joy. Each emotion is represented with color photos of four different models with nine intensity levels from 20 to 100%. The different emotional expression intensities were obtained using a morphing technique blending the neutral and maximal expression photographs for each emotion and model. The 54 photographs are organized in six lists. The lists are presented in a randomized order.

For each photograph, the task is to select the right emotion from a six-label list: fear, sadness, contempt, anger, joy, and disgust. Each photograph is presented for 10 s; there is no time limit to answer.

The total score is the percentage of correctly labeled emotions. In a previous study (49), we demonstrated that the average score of correct answers in a first population of control subjects was 76.45% (SD = 7.44).

Participants enrolled in this study all presented facial emotion recognition impairment, confirmed by a TREF score below or equal to 69% (SD = 1 below the mean of the reference population).

#### Secondary Outcome Measures

##### Clinical Assessment

Symptoms were assessed with the PANSS (51). Total, positive subscale, and negative subscale scores were used.

Delusional beliefs were assessed with the 21-item Peters et al.’s Delusions Inventory (PDI21) (54). Total scores were used.

##### Functional and Subjective Assessment

Insight was assessed with the Insight Scale (IS) (55). Total scores were used.

Self-esteem was assessed with the Self-Esteem Rating Scale (SERS) (56, 57). Total scores were used.

Social functioning was assessed with the social autonomy scale (“Echelle d’Autonomie Sociale”-EAS) (58). Total score, “social relatedness (SR),” and “relationship with the environment” (RE) sub-scores were used.

##### Cognitive Assessment

A comprehensive neuropsychological assessment of neurocognition and social cognition (see Table 2) was performed.

**Table 2:** **Comprehensive cognitive assessment**.

Neurocognition Assessment	Social cognition Assessment
Visuospatial memory: brief visual memory test – revised (59)/scores: total recall; delayed recall; recognition	Attributional style: Ambiguous Intentions Hostility Questionnaire (60)/scores: ambiguous situations HB; AB; total score
Working Memory: –Digital span (61)/Raw score –TAP (62) – Working memory – level 3/scores: TR; errors; omissions –Corsi blocks (63)/Raw score	Emotion perception: –TREF (49)-main criteria –Levels of Emotional Awareness Scale (64)/total score
Selective Attention: –D2 (65)/scores: GZ; F%; KL –TAP (62) – divided attention- visual modality; auditory modality; combined modality/scores: TR; errors; omissions	Empathy: Questionnaire of Cognitive and Affective Empathy (66)/cognitive and affective scores
Executive functions: –Trail Making Test (67)/B-A score –Key search from BADS (68)/Raw score –Verbal fluency (69)/Raw score	Theory of Mind: –Reading the mind in the eyes test (70)/total score –Versailles-Situational Intention Reading (71)/total score –Hinting task (72)/total score
Processing speed: Code (61)/Raw score

*TAP: Test of Attentional Performance; BADS: Behavioral Assessment of the Disexecutive syndrome*.

### Statistical Analysis

Analyses of variance and *t*-tests were used to investigate group differences. When results were significant (*p*-value inferior to 5%), Fisher’s least significant difference (LSD) was used. Data were analyzed using statistica (v.10). If significant differences existed between the groups at T1, *t*-tests were used on the change between T1 and T2, in order to compare evolution of measures.

To assess evolution on neuropsychological tests, statistical analyzes were performed for each cognitive process (visuospatial attention and memory, working memory, executive functions, selective attention, and processing speed). The scores were converted into standard notation in order to obtain an overall score per process.

The treatment effect magnitude was calculated with Cohen’s *d* effect size between T2 and T1 in each group.

### Hypothesis

A significant improvement in TREF scores was expected for GAÏA participants compared to the RECOS arm at T2. However, an improvement in facial emotion recognition performance was expected in both study arms at T2 compared to T1, since GAIA specifically targets emotion recognition and RECOS focuses on the role of attentional processes in facial emotion recognition (15, 17).

Various studies have shown the role of social cognition deficits in the generation and maintenance of the positive and negative symptoms of schizophrenia (15, 33, 73). Thus, decreased PANSS scores were expected in the GAIA arm at T2 compared to T1. According to the literature (34), this effect was not expected for RECOS participants.

No special assumption was made on the effects of each intervention on delusional beliefs measured with the PDI21.

Social cognition has been described as a mediator between neurocognition and social functioning (33). According to this model, an improving effect of the GAIA s-face intervention was expected at T2 on social autonomy in comparison with the RECOS program.

In a previous study (34), the RECOS program showed a positive effect on self-esteem. This effect may be due to metacognitive skills and verbalization techniques raising the participants’ awareness of their own resources, and to the problem-solving strategies they implement to manage their daily life. A large part of this methodology has been implemented in the GAÏA s-face program. Thus, an improvement of the participants’ self-esteem was expected at T2 in comparison with T1 in both study arms.

The literature describes neurocognition and social cognition as separate processes (74, 75). No effect was, therefore, expected on neurocognitive assessment in the GAÏA arm. By contrast, RECOS participants receiving cognitive remediation targeting selective attention were expected to show improved performances at T2 in tasks targeting this process compared to GAÏA participants.

Social cognition is defined as a set of separate processes (5). A specific effect of the GAÏA s-face intervention on every test measuring emotional processing was expected at T2 vs. T1. No effect of the RECOS program was expected on social cognition tests, apart from facial emotion recognition, as described above.

## Results

Results are presented in Tables 3–5.

**Table 3:** **Evolution in social cognition assessment**.

Social cognitive tasks	GAÏA (*n* = 18)	RECOS (*n* = 15)	GAÏA vs. RECOS		

	T2–T1 mean (*p*-value)	T2–T1 mean (*p*-value)	T1 (*p*-value)	T2 (*p*-value)	T2–T1 (*p*-value)
**Emotional processing**
TREF (main criteria)	0.0001***	0.01**	0.71	0.005**	–
LEAS	0.03*	0.09	0.03*	0.14	0.007**
**Attributional style**
AIHQ global	0.98	0.12	0.81	0.31	–
HB	0.08	0.53	0.63	0.36	–
AB	0.96	0.58	0.02*	0.07	0.69
**Theory of mind**
Hinting task	0.42	0.01**	0.17	0.05*	–
V-SIR	0.61	0.29	0.77	0.21	–
RMET	0.26	0.95	0.28	0.84	–
**Empathy**
QCAE cognitive score	0.79	0.02*	0.45	0.33	–
QCAE affective score	0.63	0.45	0.52	0.07	–

*V-SIR, Versailles-Situational Intention Reading test; RMET, Reading the Mind in the Eyes Test; LEAS, Levels of Emotional Awareness Scale; AIHQ, Ambiguous Intentions Hostility Questionnaire; QCAE, Questionnaire of Cognitive and Affective Empathy; (ns), no significant*.

Bold values indicate the significant result; *p < 0.05; **p < 0.01; ***p < 0.001.

### Main Outcome

**Figure 3:**
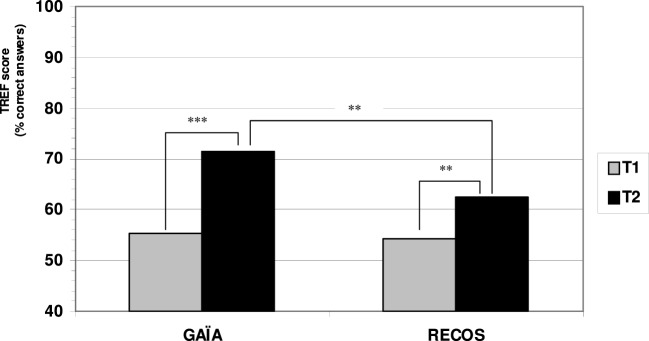
**Evolution of TREF scores pre/post intervention**.

GAÏA participants showed an average increase of 16.21% on the TREF total score at T2 compared to T1 (*p* < 0.0001), whereas RECOS participants showed an average increase of 8.43% (*p* < 0.009) for the same period. Both groups showed significantly improved T2–T1 TREF means total score (see Figure 3; Table 3).

Although there was no difference between the two groups at T1 (*p* = 0.71), a significant difference in favor of the GAÏA arm was found when comparing the results at T2 (*p* = 0.005). GAÏA participants showed significantly improved TREF scores in comparison with RECOS participants.

Each group showed a strong effect size, with a greater effect in the GAÏA arm (*d* = 2.41) than in the RECOS arm (*d* = 0.98).

### Secondary Outcomes

#### Clinical Measures

No effect was found at T2 on PDI21 measures of delusional beliefs in either group.

The GAÏA group showed significantly decreased symptoms on the T2–T1 mean (*p* < 0.001), measured with the PANSS total score. This decrease was also significant on the positive subscale score (*p* < 0.01) and the negative subscale score (*p* < 0.001).

No significant effect was found on the T2–T1 mean PANSS total score (*p* = 0.66), and positive (*p* = 0.08) and negative (*p* = 0.63) subscale scores in the RECOS group (see Table 4).

**Table 4:** **Evolution in clinical, psychosocial, and functional assessment**.

	GAÏA (*n* = 18)	RECOS (*n* = 15)	GAÏA vs. RECOS		

	T2–T1 mean (*p*-value)	T2–T1 mean (*p*-value)	T1 (*p*-value)	T2 (*p*-value)	T2–T1 (*p*-value)
**Symptoms**
PANSS TOT	0.001***	0.66	0.33	0.001***	–
PANSS POS	0.01**	0.08	0.42	0.08	–
PANSS NEG	0.001***	0.63	0.01**	0.0001***	0.066
PDI21	0.34	0.26	0.24	0.36	–
**Insight** (Birchwood)	0.09	0.95	0.01**	0.1	0.26
**Self-Estime** (SERS)	0.27	0.19	0.25	0.42	–
**Social autonomy**
EAS TOT	0.01**	0.47	0.08	0.0001***	–
RE score (EAS)	0.01**	1	0.003**	0.0001***	0.086
SR score (EAS)	0.04*	0.41	0.59	0.02*	–

*RE, relationship with the environment; SR, social relatedness*.

Bold values indicate the significant result; *p < 0.05; **p < 0.01; ***p < 0.001.

**Figure 4:**
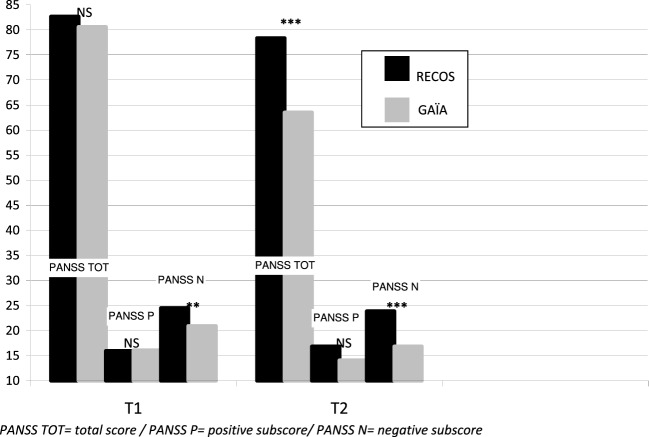
**Evolution of PANSS scores, groups comparison**.

While no significant difference was observed between the two groups in PANSS scores and sub-scores after randomization (Table 1), a significant difference in the negative sub-score appeared at T1, when taking into account drop-out participants (see Table 4 and Figure 4). Nevertheless, a significant difference in favor of the GAÏA arm was found when comparing the results at T2 on the PANSS total score (*p* < 0.001). No significant effect was found on the positive subscale between the interventions. The difference in favor of the GAÏA arm on the negative subscale is even more significant at T2 (from *p* < 0.01 to *p* < 0.0001).

#### Psychosocial and Functional Outcomes

No effect was found at T2 on measures of insight with the IS, nor on self-esteem measured with the SERS, in either group.

**Figure 5:**
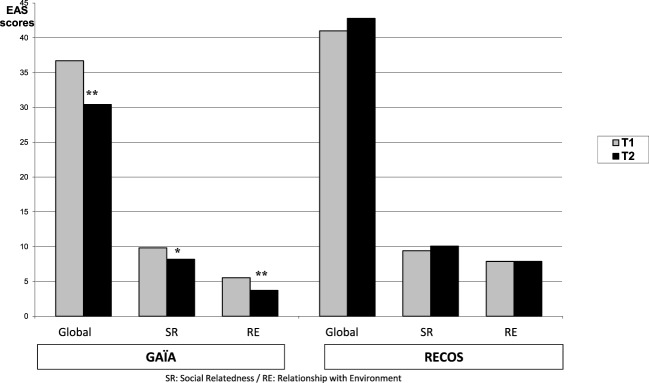
**Evolution of social autonomy, EAS scores pre/post intervention**.

Measures of social autonomy with the *EAS* significantly improved (i.e., decreased scores were observed) in the GAÏA arm on the T2–T1 mean (see Table 4; Figure 5). This improvement was found on the total score (*p* < 0.01), “SR” subscore (*p* < 0.04), and ‘‘RE’’ subscore (*p* < 0.01). No effect was found on pre–post measure in the RECOS arm.

A significant difference in favor of the GAÏA arm was found when comparing the results at T2 on the EAS total score and SR sub-score (respectively, *p* < 0.0001 and *p* < 0.02 – see Table 4). A significant difference in favor of the GAÏA arm was observed on “RE” score at T1 (*p* < 0.003); however, the difference was even more significant at T2 (*p* < 0.0001).

#### Cognitive Functioning Measures

##### Neurocognitive Assessment

Social cognitive outcomes are reported in Table 5.

**Table 5:** **Evolution in neurocognitive assessment**.

Neurocognition domain	GAÏA (*n* = 18)	RECOS (*n* = 15)	GAÏA vs. RECOS		

	T2–T1 mean (*p*-value)	T2–T1 mean (*p*-value)	T1 (*p*-value)	T2 (*p*-value)	T2–T1 (*p*-value)
Processing speed	0.03*	0.0001***	0.0001***	0.0001***	0.23
Selective attention	0.40	0.0001***	0.01**	0.05*	0.00018***
Visuospatial processes	0.001***	0.02*	0.34	0.13	
Working memory	0.01**	0.25	0.27	0.005**	–
Executive functions	0.08	0.75	0.40	0.02*	–

Bold values indicate the significant result; *p < 0.05; **p < 0.01; ***p < 0.001.

A group effect was found in selective attention on the T2–T1 mean. The participants of the RECOS arm showed significantly improved performances at T2 (*p* < 0.0001). No effect was found in the GAÏA arm (*p* = 0.4).

Despite a difference in favor of the GAÏA arm at T1 (Table 5), the significant difference found at T2 was in favor of the RECOS arm.

Both groups significantly improved in processing speed, with a larger effect in the RECOS group in comparison with the GAÏA group (respectively, *p* < 0.0001 and *p* < 0.03). No effect was found in group comparison.

Participants in both groups showed significantly improved performance in visuospatial attention and memory at T2 in comparison with T1 (see Table 5), but no effect was found in group comparison.

A group effect was found in working memory at T2. GAÏA participants showed significantly improved performance at T2 in comparison with T1 (*p* < 0.01) and with the RECOS arm (*p* < 0.005).

No effect was observed in either group during executive function assessment on T2 vs. T1 (GAÏA *p* < 0.08; RECOS *p* < 0.75). Nevertheless, a significant difference in favor of the GAÏA arm was found at T2 in group comparison (*p* < 0.02).

##### Social Cognition Assessment

Social cognitive outcomes are reported in Table 3.

Emotion perception was measured with the TREF (main criteria) and the Levels of Emotional Awareness Scale (LEAS).

A group effect measured with the LEAS was found on T2 vs. T1 for GAÏA participants, whereas no difference was found in the RECOS arm. No significant difference was found in group comparison at T2, though a difference in favor of the RECOS arm existed at T1.

No significant difference was observed in either group on the T2–T1 mean in attributional style measured with the Ambiguous Intentions Hostility Questionnaire, nor in group comparison at T2.

The assessment of theory of mind included several tasks. The Hinting task scores of RECOS participants were significantly higher at T2 than T1, whereas no effect was found in the GAÏA group. A group effect was found at T2 in favor of the RECOS arm (*p* < 0.05).

No effect was found in either group on the Versailles-Situational Intention Reading test, nor on the Reading the Mind in the Eyes test.

Empathy was measured with the Questionnaire of Cognitive and Affective Empathy. A significant increase was found between T2 and T1 in the cognitive empathy sub-score for the RECOS group (*p* < 0.02). No effect was observed in either group on the affective empathy sub-score nor in group comparison.

## Discussion

The main results include improved facial emotion recognition performance in both groups, reduced symptoms and improved social functioning in the GAÏA group, and the improvement of some neurocognitive and social cognitive processes in both study arms.

The significant improvement in TREF results for the GAÏA arm compared to the RECOS arm is consistent with the data from the literature, since Kurtz and Richardson (32) already showed that facial emotion recognition could be improved using a specific remediation.

The improvement for RECOS participants was an expected effect, based on previously demonstrated links between selective attention and facial emotion recognition skills (15–17).

Various hypotheses may explain the significantly greater improvement of the GAÏA group in facial emotion recognition skills compared to the RECOS group.

The GAÏA program learns participants to focus on relevant facial features to recognize emotions; this specific training probably helped them directly reuse the strategies learned during cognitive remediation to perform the TREF test. As for the RECOS group, the ability to select and focus on relevant facial features may be considered as a transfer and generalization effect of the strategies they practiced during cognitive remediation.

Previous research has shown that modifications in the pattern of visual attention to faces were associated with facial emotion recognition deficit (20, 21). The GAÏA program probably has a positive effect on the pattern of visual attention to faces, supported by an (unexpected) positive effect on visuospatial abilities on pre–post analysis. However, further studies should explore this hypothesis.

The significant improvement of schizophrenia symptoms measured with the PANSS at T2 in the GAÏA arm was expected. Various studies have shown correlations between social cognition, especially facial emotion recognition, and both positive (15, 73) and negative (33) symptoms.

However, links between symptoms and social cognition are not straightforward (33), since reduced symptoms after GAÏA s-face therapy may be a transfer and generalization effect of the benefits of cognitive remediation.

Symptoms did not improve at T2 in the RECOS arm, hypothetically because of a lack of connection between neurocognition and positive symptoms (76), unlike social cognition that may act as a mediator between neurocognition and negative symptoms (33). The improvement of negative symptoms after neurocognitive remediation, thus, constitutes a higher transfer level than facial emotion recognition.

Nevertheless, it will be important to observe whether these benefits are sustainable and whether a generalization effect occurs in the RECOS arm after follow-up, as it was shown in a previous study (34).

A significant improvement in social autonomy was measured with the social autonomy scale (EAS) (58) for GAÏA participants. Such improvement is consistent with the data from the literature, which describe social cognition as a mediator between neurocognitive and social functioning (77, 78). Couture et al. specifically showed correlations between the perception of emotions and community functioning, “social behavior in the milieu” and “social skills,” which seems to match the improvement in the ‘‘relationship with the environment’’ (RE) and ‘‘social relatedness’’ (SR) subdivisions of the EAS. We suggest that this effect was encouraged by individual therapy, which helped introduce a metacognitive dimension to the sessions with the therapist, and adapt exercises to the motivations and clinical profile of participants during the transfer sessions. Furthermore, the structure of the GAÏA s-face program, offering computer exercises adjusted to the participant’s everyday environment, may have facilitated the transfer and generalization of benefits to everyday life.

This study did not show any improvement in social functioning for RECOS participants, probably because transfer between neurocognition and social functioning is less straightforward than between social cognition and social functioning. However, the program is open to incorporate a metacognitive dimension and individualization, to facilitate the transfer of strategies to daily tasks.

It will be important for future studies to observe how social functioning evolves in the long term for both programs, whether the encouraging results of the GAÏA arm are long-lasting, and whether functional benefits appear later for the RECOS arm, since the generalization of cognitive benefits requires a certain amount of time.

An improvement in self-esteem was expected in both arms, but this study did not evidence this improvement. RECOS had been proven effective in this area in a previous study (35) related to therapeutic strategies, particularly the research and generation of the participant’s own strategies and the positive reinforcement provided by the therapist. These strategies were introduced into the GAÏA s-face program. It will be important for future studies to perform the same measures with more experienced therapists and assess subjective benefits for the participants as well.

This study also highlighted the benefits of cognitive remediation with the RECOS program. The RECOS group underwent a training program focusing on selective attention (in its usual version, the program provides targeted cognitive remediation, adapted to the participant’s cognitive profile) and results at T2 showed a significant improvement in selective attention, processing speed, and visuospatial attention tasks.

The GAÏA group also showed positive effects on neuropsychological assessment at T2. Besides visuospatial functions, improved working memory was observed after the therapy. Several hypotheses may explain this unexpected effect. Some neuropsychological tests measuring working memory skills used visual modality and, therefore, also required visuospatial memory skills. Improvement in memory and visuospatial attention for GAÏA participants may underlie improvement in working memory.

Furthermore, the GAÏA s-face therapy requires that participants undertake verbal and visual learning, especially during photograph exercises and generalization phases, where they have to assimilate cues to recognize facial expressions of basic emotions. Therapists probably developed strategies with participants to facilitate these acquisitions and participants may have reused such strategies during T2 working memory tests. However, it will be necessary to repeat these results in future studies designed to test these hypotheses.

The assessment of social cognition evolution at T2 in the GAÏA arm showed a significant improvement in the participants’ performance in tests measuring emotional perception. Because of the specific nature of the LEAS (66), the significantly increased scores in this test could be a first step toward improved empathy skills; however, no improvement was found in this area.

Nonetheless, this targeted increase seems in accordance with the literature, which defines social cognition as a complex process with partially independent components (6, 79).

A significant improvement was observed in one of the theory of mind tests [the Hinting task (72)] and in cognitive empathy measured with the QCAE for RECOS participants. The Hinting task assesses the ability to understand implicit components within sentences from daily life situations; the QCAE (cognitive subscore) measures a person’s ability to consider a situation from someone else’s perspective. Such improvement may be partly explained by improved attention skills, enabling participants to better focus on situations rather than on their own thoughts or feelings, and better detect intentions in verbal messages.

Limitations of the present study mainly include small sample size and lack of evaluation of the long-term preservation of the benefits.

Further studies are needed to confirm the positive outcomes of the present study and verify their sustainability.

The potential impact on the subjective quality of life of the participants should be assessed in future studies, using standardized assessment and real-life social functioning criteria.

Finally, further studies will have to explore the effects of the GAÏA s-face program on every social cognitive process (social perception and social knowledge were not assessed in this study). Comprehensive social cognition test batteries are currently under development (80); they should help better understand the interactions between each process.

## Conclusion

Although the understanding of the cognitive mechanisms improved by the GAÏA program is still incomplete, the results of this study show the applicability and effectiveness of the program to improve facial emotion recognition skills, reduce positive and negative symptoms in schizophrenia, and improve social functioning.

Positive results were obtained for both programs in the main criteria. Facial emotion recognition skills improved in both arms, selective attention was significantly improved in the RECOS group, which received specific training targeting these processes, and the GAÏA program showed positive effects on symptoms and social functioning. These results were obtained with cognitive remediation provided by nurses after a 3-day training session. This is an interesting prospect for the spread of cognitive remediation and its potential inclusion into the routine care of schizophrenia alongside drug treatment, psychoeducation, social skills training, and psychotherapy.

Facial emotion processing is also impaired in other disorders, so it would be interesting for future studies to explore whether the GAÏA s-face program is effective in people with diseases, such as autism spectrum disorders or 22q11.2 deletion syndrome.

## Author Contributions

BG: nurse; GAÏA s-face program conceptor; contibution in study design; and first author. JV: neuropsychologist; cognitive assessment; contibution in study design; DATA analysis; substantial contribution. SG: nurse; symptoms, social functioning and subjective assessment; contribution in study design; substantial contribution. The GAÏA/RECOS study team: Dupuis M., Hochard C., Josserand AC., Koubichkine A., Lambert T., Perez M., Rouyre B.: nurses; therapists; critical review. Scherding P.: occupational therapist; therapist; critical review. Bralet MC., Demily C., Launay C., Gouache B: medical investigators; critical review. Duboc C., Dubrulle A., Farhat SL., Fourt A., Fluttaz C., Peyroux E., Todd A.: neuropsychologist assessors; critical review. Prof. NF (last author): first investigator; substantial contribution in GAÏA s-face program conception; substantial contribution in study design; substantial contribution in writing the article.

## Conflict of Interest Statement

The authors declare that the research was conducted in the absence of any commercial or financial relationships that could be construed as a potential conflict of interest.
